# Understanding Spatial Genome Organization: Methods and Insights

**DOI:** 10.1016/j.gpb.2016.01.002

**Published:** 2016-02-11

**Authors:** Vijay Ramani, Jay Shendure, Zhijun Duan

**Affiliations:** 1 Department of Genome Sciences, University of Washington, Seattle, WA 98195, USA; 2 Institute for Stem Cell and Regenerative Medicine, University of Washington, Seattle, WA 98109, USA; 3 Division of Hematology, University of Washington, Seattle, WA 98195, USA; 4 Department of Medicine, University of Washington, Seattle, WA 98195, USA; 5 Howard Hughes Medical Institute, Seattle, WA 98195, USA

**Keywords:** Chromatin, Chromosome, Epigenomics, 4D nucleome, Hi-C

## Abstract

The manner by which eukaryotic genomes are packaged into nuclei while maintaining crucial nuclear functions remains one of the fundamental mysteries in biology. Over the last ten years, we have witnessed rapid advances in both microscopic and nucleic acid-based approaches to map genome architecture, and the application of these approaches to the dissection of higher-order chromosomal structures has yielded much new information. It is becoming increasingly clear, for example, that interphase **chromosomes** form stable, multilevel hierarchical structures. Among them, self-associating domains like so-called topologically associating domains (TADs) appear to be building blocks for large-scale genomic organization. This review describes features of these broadly-defined hierarchical structures, insights into the mechanisms underlying their formation, our current understanding of how interactions in the nuclear space are linked to gene regulation, and important future directions for the field.

## Introduction

The human body consists of many trillions of cells harboring nearly identical genomes, and yet subsets of these cells are distinct both functionally and morphologically. It is widely accepted that “epigenetic” mechanisms are responsible for the differential regulation of shared genetic information, and thus for the generation of a diverse array of terminal cell types through zygotic development.

The physical organization of eukaryotic chromosomes within a nucleus is crucially intertwined with the reading, interpretation, and propagation of genetic information by these epigenetic mechanisms. Metazoan cells package genomic DNA up to 2 m long into a tiny nuclear space ∼10 μm in diameter via hierarchy of organizational structures [1]. The compaction begins with the wrapping of 147 base pairs (bp) of DNA around a histone octamer to form the nucleosome; this nucleoprotein complex serves as the basic repeating unit of chromatin. The histone octamer itself is composed of eight subunits that assemble as one histone H3–H4 tetramer and two histone H2A–H2B dimers. Both DNA and histone components of the nucleosome particle can be subjected to a diversity of chemical modifications [2] (*e.g.*, CpG methylation and lysine tail acetylation), and several histone variants exist [3] (*e.g.*, H2A.Z, CENPA), thus enabling an epigenetic diversity at even this most basic level of chromatin organization. The next level of compaction is commonly believed to be the organization of nucleosomes into a 10 nm “beads-on-string” chromatin fiber [4]. Additional nucleosomal organization into higher-order structures on the order of 30 nm or 100 nm has been hotly debated, and the existence of native structure beyond the 10 nm fiber has been questioned [5], [6]. For instance, a recent study has proposed that native chromatin fibers in *Saccharomyces cerevisiae* are formed by heterogeneous clutches of nucleosomes that are interspersed with nucleosome-depleted regions, arguing against the existence of highly-ordered structures such as the 30 nm fiber [7]. Regardless of physical model, however, the chromatin fiber must ultimately fold into highly-condensed interphase chromosomes, a process that remains poorly understood.

Though we still know little of the dynamics of *in vivo* chromatin folding, we have gained important insights into the higher-order spatial organization of eukaryotic genomes, thanks to significant advances in DNA imaging technology and high-throughput biochemical techniques [8], [9], [10], [11]. Eukaryotic genomes are clearly organized in the nucleus in a nonrandom way. In mammalian genomes, individual chromosomes preferentially occupy distinct nuclear areas, termed chromosome territories (CTs) [12]. Transcriptionally-silent regions are generally localized near the nuclear envelope and perinucleolar space, whereas transcriptionally-active regions occupy the remaining nuclear space [13], [14]. At the cytological level, the eukaryotic genome is partitioned into euchromatin and heterochromatin [1]. At the molecular level, the nucleus is geometrically compartmentalized in mammalian cells to contain morphologically and molecularly distinct sub-structures (*e.g.*, nuclear bodies), suggesting that nuclear activities are also spatially organized [15], [16]. Individual chromosomes are partitioned into various physical or functional compartments and domains such as topologically associating domains (TADs) and chromatin loops [10], [17], [18], [19]. Given the strong link between these common organizational features and cellular functions (*e.g.*, transcription), it is tempting to speculate that the modulation of chromatin organization itself is a basic mechanism by which cellular functions are enacted. However, crucial experiments, some of which are mentioned later in this review, are still required to elucidate whether these observed structural features play some general causal roles, or are simply a correlative of the cellular functions. Even still, we can be reasonably certain that features like chromatin loops, clustered highly-transcribed genomic loci, and large-scale chromosome domains are basic elements of chromatin folding, and as such are invaluable when analyzing genome organization in the context of developmental and environmental cues.

Here, we first review well-established and emerging technologies that are revolutionizing our understanding of higher-order genome architecture. We then discuss our current understanding of spatial genome organization in greater detail, providing insights into the mechanisms underlying structure formation, and the links between chromatin folding and gene regulation. Finally, we propose a handful of pressing questions we believe to be central to an ultimate understanding of the spatiotemporal organization and functions of nucleome.

## Tools for exploring the 3D genome

Three largely-orthogonal approaches are commonly used to study the structure and function of the three-dimensional (3D) genome. Microscopy-based DNA imaging techniques and high-throughput genomic mapping tools based on massively-parallel sequencing have been used to delineate higher-order genomic architecture, while genome perturbation tools (*i.e.*, genome-editing) have then been used to ascertain the functional significance of specific architectural elements. Over the last ten years, the field has witnessed tremendous methodological advances in all three areas [20], [21], [22], [23].

Traditionally, chromosome and nuclear structure have been viewed through DNA imaging technologies, which can be based on electron microscopy [24], [25], or light microscopy [25], [26]. Electron microscopic techniques, including transmission electron microscopy (TEM) and cryo-electron microscopy (Cryo-EM), have typically been used to characterize cell-free systems. Cryo-EM, in particular, has become an increasingly popular structural biological tool, owing in part to dramatic improvements in resolution and ease of sample preparation [27]. Recently, Cryo-EM was used to determine an 11 Å-resolution structure of 30-nm chromatin fibers assembled from arrays of 12 nucleosomes [28].

Before the advent of massively-parallel analyses by microarray and later high-throughput sequencing, our knowledge of 3D genome organization was largely derived from studies using fluorescence labeling-based light microscopy, such as DNA fluorescence *in situ* hybridization (FISH) [29] and live-cell imaging [30]. FISH and live-cell imaging can directly measure physical distances between DNA loci and visualize the nuclear position of loci and/or whole chromosomes within single cells. Today, many variants of the FISH technique exist, including conventional two-dimensional FISH (2D-FISH), 3D-FISH [31], and cryo-FISH [32], with the resolution approaching 100 kb [33]. More recently, a high-throughput imaging position mapping platform (HIPmap) has been implemented [34], presenting a crucial breakthrough in overcoming the limitations of scalability and throughput that are associated with conventional FISH techniques. While FISH assays are typically used to characterize only a few loci at a time, HIPmap enables large-scale (384-well format), automated, high-resolution localization of 3D gene positions in single cells. In addition to HIPmap, a quantitative high-resolution imaging approach, which combines FISH, array tomography (AT) imaging, and multiplexed immunostaining, has also been implemented for investigating 3D chromatin organization in complex tissues [35]. The development of the automated image analysis toolkits such as the aforementioned ones is likely to be critical as the field moves toward visualizing chromatin architecture in a large number of diverse contexts.

One commonly-cited limitation of light microscopic techniques, despite their versatility, is the resolution limit owing to the wavelength of light. To overcome this, several super-resolution fluorescence microscopy approaches, such as structured illumination microscopy (SIM), stimulated emission depletion (STED), and photoactivation localization microscopy/stochastic optical reconstruction microscopy (PALM/STORM), have been developed during the last decade (reviewed in [36]). These techniques have been applied to study higher-order nuclear architecture [37], [38], [39], [40]. In addition, combined with more advanced fluorescent labeling techniques, these techniques have also been used to image chromosome dynamics with unprecedented spatiotemporal resolution in live cells at the single-molecule level [41]. In typical chromatin visualization experiments, either chromatin-associated proteins (*e.g.*, core histone proteins) or the DNA itself must be labeled [37]. While the LacO/LacI DNA tagging system has long been used in live-cell imaging [14], recent developments in live-cell chromatin imaging have used fluorescently-tagged transcription activator-like effector (TALE) proteins [42] or the clustered regulatory interspaced short palindromic repeat (CRISPR)/Cas9 system [43], [44] to specifically label loci.

Complementary to microscopy-based DNA imaging tools, biochemical tools decipher nuclear organization by measuring physical contacts between different genomic regions or between genomic DNA and other nuclear components. Initially coupled with oligonucleotide-decorated array (microarray) technology, and now typically paired with massively-parallel DNA sequencing [45], these biochemical tools enable genome-wide characterization of myriad aspects of higher-order chromosome structure and organization. Moreover, they can also allow for reconstruction and modeling of 3D genome architecture with the aid of sophisticated computational algorithms [46].

**Figure 1:**
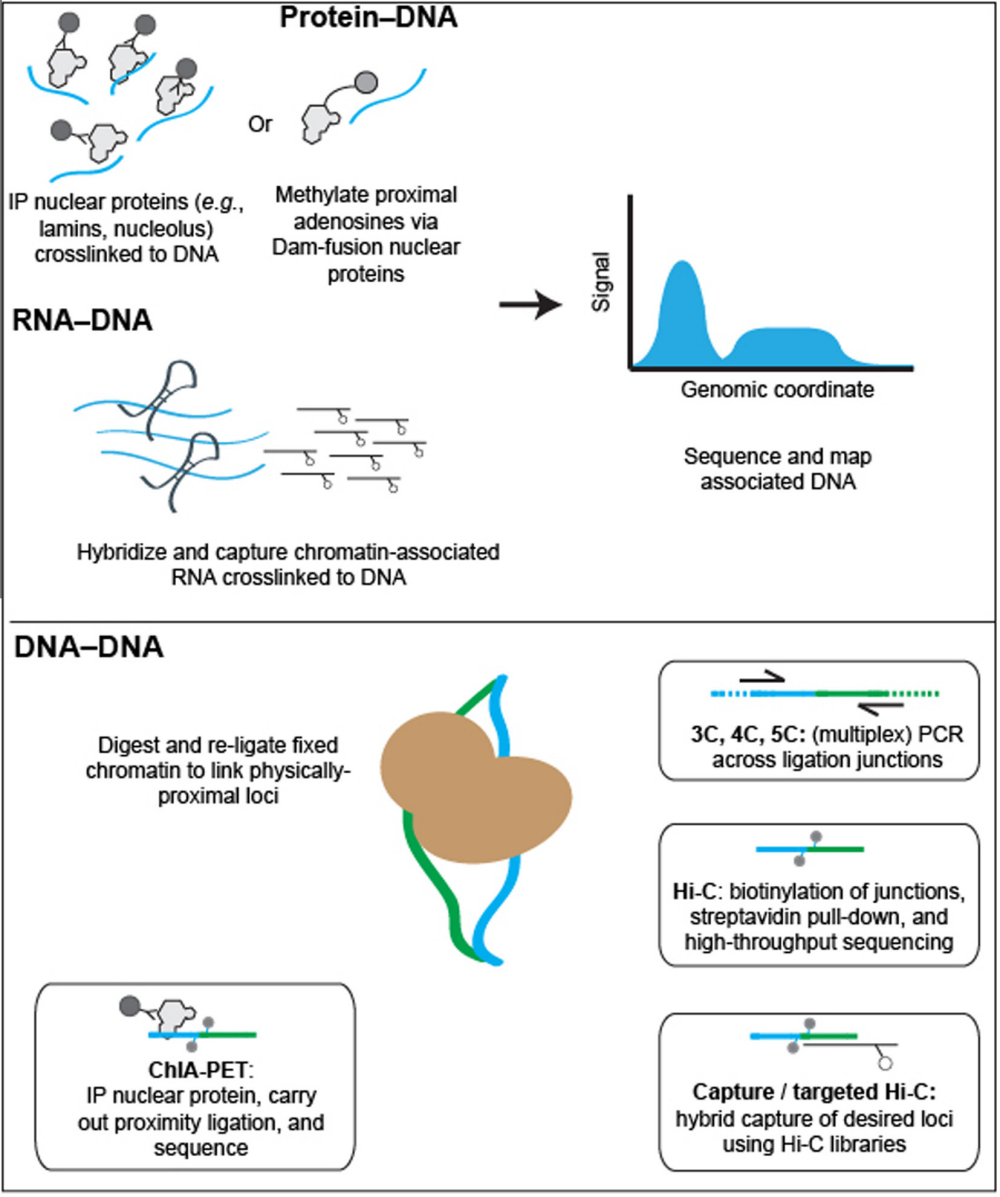
**High-throughput biochemical techniques for probing the nucleome** High-throughput methods for probing the nucleome can broadly be grouped into three classes. (1) Methods detecting protein–DNA interactions include ChIP-seq, where antibodies specific to proteins of interest are used to co-precipitate crosslinked genomic DNA, and DNA adenine methyltransferase identification (DamID), in which a bacterial adenine methylase is used to methylate physically-proximal adenines. (2) Methods detecting RNA–DNA interactions include ChIRP, CHART, and RAP. Crosslinked chromatin is sheared and then hybridized to biotinylated anti-sense oligonucleotides specific to a transcript or transcripts of interest. In all of these methods, tagged or purified DNA is used to create a massively-parallel sequencing library. (3) The 3C family of methods are used to probe DNA–DNA interactions. While there are many different types of 3C assay, all 3C-based methods share the same core concept: chromatin interactions are measured by proximity ligation of fragmented and crosslinked chromatin. The key differences between these methods lie in how chromatin interactions are detected following proximity ligation. In ChIA-PET, crosslinked chromatin complexes are fragmented by sonication and chromatin interactions mediated by a protein of interest are enriched by ChIP before performing the proximity ligation. In 3C, 4C, and 5C, chromatin interactions of interest are enriched by PCR using locus-specific primers. In Hi-C and its variants, the valid chromatin interactions are enriched through a streptavidin-biotin-mediated pull-down. In targeted Hi-C methods, such as Capture-C, Capture Hi-C, and targeted DNase Hi-C, chromatin interactions of interest are enriched by applying hybrid capture technologies to 3C or Hi-C libraries.

Broadly, the current state-of-the-art for these biochemical techniques can be classified into three groups, based on the biological origin of the chromatin contacts being assayed (Figure 1; Table 1). Methods that can detect DNA-protein interactions, such as chromatin immunoprecipitation (ChIP-seq), DNA adenine methyltransferase identification (DamID), and sedimentation fractionation, have been used for probing physical contacts between genomic loci and nuclear landmarks such as the nuclear envelope or nucleolus, providing information about where particular genomic loci are localized within the nucleus. In ChIP techniques [47], antibodies specific to a nuclear complex of interest are used to immunoprecipitate chemically-crosslinked sheared chromatin, and the associated DNA is used to create a high-throughput sequencing library. In DamID [48], bacterial adenine methyltransferase is fused to a protein of interest and allowed to interact with physically-proximal DNA. Sequences containing methylated adenine are enriched through digestion with Dam-specific restriction enzymes, and the products are then sequenced. In sedimentation fractionation [49], chromatin is subjected to ultracentrifugation and fractionation, and the DNA present in desired fractions is sequenced. Both ChIP and DamID have been used to identify genomic regions associated with nuclear pore complexes (NPCs) [20], while sedimentation fractionation has been used to isolate nucleolus-associated domains (NADs) [49]. Most commonly, the DamID approach has been used to catalog genomic regions that interact with the inner face of nuclear membrane, the so-called lamina-associated domains (LADs) [50], [51], [52].

The second class of methods includes those that probe chromatin–RNA interactions, a hotly debated class of interactions that may eventually be used to define chromatin domains or sub-nuclear bodies. Currently, there are three different methods for identifying chromatin–RNA interactions: chromatin isolation by RNA purification (ChIRP) [53], capture hybridization analysis of RNA targets (CHART) [54], and RNA antisense purification (RAP) [55]. All three techniques follow the same basic schema: crosslinked chromatin is sheared and then hybridized to biotinylated anti-sense oligonucleotides that are specific to a transcript or transcripts of interest. Following a streptavidin enrichment step, DNA co-enriched with targeted RNA is subjected to deep sequencing. All three of these techniques have been used to study long noncoding RNAs (lncRNAs), including RoX in *Drosophila melanogaster* [56] and Xist in mouse [54], [55], both of which play crucial roles in each species’ respective dosage compensation mechanisms.

The third group of techniques covers the chromosome conformation capture (3C) family of methods [21], [57], which measure the relative spatial proximity between individual genomic loci through digestion and re-ligation of physically-proximal chemically-crosslinked fragments of chromatin. 3C techniques are probably the most popular tools for mapping chromatin interactions, and a diversity of methods based on 3C have been developed during the past decade. 3C derivatives themselves can be classified into two groups (Table 2): (1) for globally mapping genome-scale chromatin interactions occurring in a nucleus, including Hi-C [58], tethered conformation capture (TCC) [59], single-cell Hi-C [60], DNase Hi-C [61], *in situ* Hi-C [62], *in situ* DNase Hi-C [63], and Micro-C [64]; and (2) for targeted detection of a subset of chromatin interactions, such as 3C [57], ChIP-loop [65], circularized chromosome conformation capture (4C) [66], [67], enhanced 4C (e4C) [68], carbon-copy chromosome conformation capture (5C) [69], chromatin interaction analysis by paired-end tag sequencing (ChIA-PET) [70], [71], Capture-C [72], Capture-Hi-C [73], and targeted DNase Hi-C [61]. Since 2009, Hi-C and its variants have been used to generate whole-genome contact probability maps in bacteria [74], [75], [76], budding and fission yeast [64], [77], [78], a pathogenic eukaryote (*Plasmodium falciparum*) [79], plants [80], [81], worm [82], fly [83], [84], mouse [63], [85], and human [58], [62], [85], [86]. Depending on the protocol and depth of high-throughput sequencing used, the resolution of Hi-C-derived contact probability maps can be multiple orders of magnitude lower than that of genomic annotations at base-pair level. In the absence of incredibly-high sequencing depth, Hi-C and its variants are most suitable for identifying chromatin conformation signatures at the sub-megabase (Mb) or Mb scale, such as CTs, chromatin compartments, and TADs. Other chromatin conformation signatures, including so-called loops between promoters and other *cis*-elements, or pairs of binding sites for the transcription factor CTCF, are best carried out using the second group of approaches, which are each designed to map a specific set of chromatin interactions and thus allow for considerably-higher resolution for a given sequencing depth. Indeed, 4C, 5C, ChIA-PET, Capture-C, Capture-Hi-C, and targeted DNase Hi-C have all successfully been used to map specific regulatory interactions.

Crucially, data generated using 3C technologies may also be used to generate 3D predictions of genomic structure. The various computational approaches for tackling this problem have been reviewed in great detail elsewhere [87], and as such this review will not further address this large and still growing body of work.

The biochemical methods discussed above are able to offer detailed molecular views of chromosome structure. However, these assays are all performed on many thousands to millions of cells per experiment, thus masking the variability inherent between individual cells. Single-cell versions of ChIP-seq [88], Dam-ID [52], and Hi-C [60], [89] have all been recently described, though in all cases the sensitivity of the assay is markedly low due to the difficulty in obtaining large amounts of DNA from single cells. Still, this field of single-cell chromatin profiling by high-throughput biochemical methods is nascent, and offers an interesting complement to traditional single-cell assays carried out through microscopy. The most accurate models for the spatiotemporal organization of eukaryotic genome architecture will likely be derived using a combination of high-resolution microscopy-based imaging technologies (FISH and live-cell imaging) and high-throughput, genome-wide single-cell biochemical approaches.

A major goal in the field of chromatin biology concerns characterizing the functional significance of the genomic regions identified using the aforementioned techniques. Several genome editing tools are currently available, including the zinc-finger nucleases (ZFNs) [90], transcription activator-like effector nucleases (TALENs), and the RNA-guided CRISPR/Cas9 system [22]. All of these tools have been used to perturb higher-order chromatin architecture through genome and epigenome editing [91], [92], [93], [94], [95], [96], [97], [98]. Due to limited space, this review will not cover these tools and their applications, which have been reviewed elsewhere [99].

## Organizational features of eukaryotic genomes and their relation to nuclear activities

Microscopy-based and high-throughput biochemical studies have revealed common organizational structures in eukaryotic genomes, including CTs, chromatin, and nuclear compartments, various types of chromatin domains (*e.g.*, NADs, LADs, and TADs), and chromatin loops. In this section, we discuss their respective biophysical characteristics, and links between these structural features.

### CTs and the nuclear position of chromosomes

The non-randomness of genome organization in the nuclear space at chromosome level was observed more than a century ago. The Rabl configuration, with centromeres and telomeres at opposite poles of the nucleus, was proposed by Carl Rabl in 1885 [100] and later confirmed by both microscopic and molecular studies in yeast and some plants [77], [80], [81]. In 1909, Theodor Boveri suggested that animal interphase chromosomes occupied distinct regions within the nucleus, for which Boveri introduced the term CTs. Since then, microscopic studies and genome-wide chromatin interaction mapping have revealed several features of CTs. First, although the existence of CTs in yeast and some plants is debatable, CTs as an organizational feature exist in the nuclei of a wide range of species, particularly mammals [85], [86]. Second, each CT is predominantly a self-interacting entity that still harbors interactions with other CTs [12]. The physical clustering of centromeres, ribosomal DNA (rDNA) genes, and tRNA genes located on different chromosomes, which can be seen in species as divergent as *S. cerevisiae* and human, is a prime example of contacts occurring between different CTs [58], [59], [77], [83], [84]. Third, although the position of each CT is stochastic in a cell population (*i.e.*, not the same in each cell), individual CTs show preferences for nuclear positioning in mammalian cells, which may correlate with genomic properties (*e.g.*, GC content, gene density, and chromosome size), as well as with genomic functions (*e.g.*, transcriptional activity and replication timing) [101], [102], [103], [104], [105]. In general, large and gene-poor chromosomes tend to be located near the nuclear periphery, whereas small and gene-rich chromosomes group together near the center of the nucleus. For example, human chromosomes 18 (gene-poor) and 19 (gene-rich) are localized preferentially to the periphery and center of the nucleus in human lymphocytes, respectively [102]. Interestingly, homologous chromosomes in diploid cells are generally found to be far apart from each other in the interphase [106]. Fourth, in each cell, the relative position of CTs is stably maintained from mid G1 to late G2/early prophase during the cell cycle; this has been demonstrated in both HeLa cells and normal rat kidney (NRK) cells [107], [108]. Whether these global chromosomal arrangements are transmitted through mitosis, however, remains unknown. In NRK cells, this is believed to be the case [107], while in HeLa and HT1080 fibrosarcoma cells, this appears to not be the case [108], [109]. Fifth and the last, while the functional significance of a given CT’s positional preference remains unknown, the spatial configurations of chromosomes relative to one another are tissue-specific [110] and may even be evolutionarily-conserved [111]. As an example of tissue specificity, X chromosomes are localized more peripherally in liver cells compared to kidney cells [110].

### Chromatin folding and compartmentalization of nuclear activities

At any given time within a living cell’s interphase chromosomes, certain genomic loci may be embedded in a constitutive heterochromatin region, some may associate with the nuclear lamina, some may be attached to the nucleolus, and others may be embedded in the various sub-nuclear bodies, engaging in specific nuclear activities. One widely-held model for transcription postulates that active genes may co-localize into discrete “transcription factories”, where high local concentrations of RNA polymerase II (RNAPII) and basal transcriptional machinery enforce gene expression [112]. Thus, in any given nucleus of a eukaryotic cell, along an interphase chromatin fiber, packing state is heterogeneous and tightly associated with local epigenetic state. This supports the notion that chromatin folding is somehow influenced by various nuclear processes (*e.g.*, transcription and DNA replication/repair) and constrained by nuclear context (*e.g.*, geometrical heterogeneity). Microscopic and molecular studies have identified several chromatin domains, with each representing some aspect of chromatin folding. Here we summarize the characteristics of the most commonly-discussed chromatin domains and review how they relate among each other.

### A/B compartments

Hi-C studies have revealed that within CTs, chromosomes are partitioned into large compartments at the multi-Mb scale, containing either the active and open (A compartments) or inactive and closed chromatin (B compartments) [58]. The open A compartments contain high GC-content regions, are gene-rich, and are generally highly transcribed. They are enriched in DNase I hypersensitivity and histone modifications marking active (H3K36me3) and poised chromatin (H3K27me3). In contrast, B compartments are gene-poor, less transcriptionally active, and enriched in high levels of the silencing H3K9me3 mark [58]. It is interesting to consider the extent to which A/B compartments are correlated with cytogenetically-defined euchromatin/heterochromatin. The A compartments preferentially cluster with other A compartments throughout the genome, as do B compartments. B compartments are also highly correlated with late replication timing and LADs, suggesting that their nuclear position might be close to the nuclear periphery [113]. A recent high-resolution Hi-C study found that the two compartments can be further subdivided into six sub-compartments (A1, A2, and B1-B4) [62]. A/B compartments and sub-compartments have also been found to be cell-type specific and are each associated with distinct chromatin patterns [58], [62]. This represents, a sensible finding given that different cell types express gene sets driven by distinct groups of regulatory elements. Thus, the compartmentalization of CTs into distinct A/B compartments and sub-compartments is directly correlated with the cell type-specific gene expression and chromatin status of the genome. Indeed, A/B compartments revealed by Hi-C can be reconstructed by using a variety of epigenomic data, reflecting genome-wide DNA methylation or chromatin accessibility patterns [114].

### Self-interacting domains

With increases in resolution provided by a greater depth of sequencing, recent Hi-C and 5C studies have revealed that CTs and A/B compartments may be broken down further into smaller self-interacting domains, which have been identified in the genomes of a wide range of species from bacteria to human [115], [116]. In metazoan genomes, these chromatin-folding modules are called physical domains in flies [84] or TADs in mammalian cells [85], [117], while in bacteria and yeast, these domains are typically referred to as chromosomal interacting domains (CIDs) [64], [74]. TADs in mammalian genomes are several hundred kb up to 1–2 Mb in size (with a median size of about 800 kb in mouse) [85], [117], and are smaller in flies (60 kb) [83], [84], while CIDs are typically smaller [64], [74].

While the formal definition for these self-interacting domains is quite broad, they all share common core properties. First, they are characterized by a greater frequency of within-domain interactions as compared to external interactions. This is in fact how TADs are identified in Hi-C data, through a measure of the directionality index (DI) of ligation pairs across a chromosome [85]. Identification of self-interacting domains is thus strongly dependent on the resolution of the Hi-C data set analyzed. This is evidenced by the much smaller self-interacting domains (median length 185 kb), identified in both mouse and human cells in a recent high-resolution Hi-C study [62]. Second, domain boundary regions are generally enriched in transcription start sites, active transcription, active chromatin marks, housekeeping genes, tRNA genes, and short interspersed nuclear elements (SINEs), as well as binding sites for architectural proteins like CTCF and cohesin [85]. A recent study also highlighted the role of histone acetylation in the formation of TADs, suggesting that TADs are primarily built from nonacetylated nucleosomes and that TAD boundaries are composed of acetylated nucleosomes [118]. Third, TADs are evolutionarily conserved and cell-type independent [115], [116], a feature that is expected, given the presence of housekeeping genes at TAD boundaries. Fourth, self-interacting domains represent basic units of chromatin folding. This is supported by early microscopic studies showing that CTs consist of chromosomal domains (CDs) spanning 100 kb–1 Mb in size [119], the same length scale as for the recently defined self-interacting domains. This suggests that TADs and similar domains may represent the same structures as microscopy-defined CDs. Recent lines of evidence further strengthened this by linking TADs and chromatin packing directly in fly [120]. Hi-C studies on *Drosophila* polytene chromosomes revealed equivalence between polytene bands/inter-bands and TAD/TAD boundaries, suggesting that different types of TADs correspond to distinct packing states. For example, inactive TADs, which contain fully-condensed chromatin at the nuclear periphery, correspond to classical heterochromatin, whereas active TADs (partially-packaged) and TAD boundaries (fully-extended chromatin fibers) correspond to classic euchromatin (less dense chromatin in the nuclear interior). Since these polytene bands are observed in single salivary gland cells, the correspondence of TADs to polytene bands also suggests that TADs are unlikely to be a statistical feature of population-level Hi-C experiments, but rather exist at the level of single cells. Recently, a super-resolution microscopy study on human and mouse cells using STORM revealed that nucleosomes are grouped into discrete clutches along the fiber, with areas of relative depletion between them [7]. The relationship between these “clutches” of nucleosomes and self-interacting domains in metazoans remains unknown, though the recently published Micro-C method in yeast hints at a strong linkage between the two [64].

Though the definition of self-interacting domains has greatly helped our understanding of how chromatin might be organized in the nucleus, the functional relevance of these domains and the mechanisms underlying their formation remain poorly understood. To get at the function of particular domain boundaries, recent studies have employed genome editing to edit out or invert CTCF sites [94], [96], [98]. In some cases, this editing led to drastic changes in gene expression, particularly when single nucleotide polymorphisms (SNPs) in these CTCF sites were already implicated in genome-wide association studies (GWAS) for a particular syndrome. Another naturally-occurring example of this was recently shown in the context of brain cancer, where hypermethylation at particular CTCF sites in low-grade *IDH1-*mutant gliomas leads to differential CTCF binding, changes in genome topology, and consequent dysregulation of proto-oncogenes [121]. In other cases, however, inversion or deletion led to only slight changes in gene expression. The results of such experiments hint at the underlying complexity of gene regulation, perhaps suggesting that genome architecture alone is not the master regulator of gene expression.

### Gene clustering in transcription factories

One common model for transcription posits the existence of transcription factories—discrete nuclear foci in eukaryotic nuclei where transcription occurs [122]. Biochemical purification of transcription factories associated with RNAPI, II, or III has demonstrated that transcription factories consist of nascent RNAs, genomic templates and regulatory DNA elements (*e.g.*, enhancers), and a variety of proteins involved in transcription initiation, elongation, and regulation [122], [123]. Several features of transcription factories have been revealed: (i) >95% of all nuclear transcription activities occur within transcription factories [124]; (ii) each transcription factory contains only one type of RNAP (I, II, or III) and the number of the RNAP molecules in a factory is variable among different cell types [124]; (iii) genes sharing the same factory can be on the same chromosome or on different chromosomes, and may be co-regulated or functionally unrelated [125]; (iv) the number of transcription factories found per nucleus depends largely on the species studied and the detection method used, ranging from a few hundreds to a few thousands [124]; and (v) the size of the factory varies depending on both the RNAP featured and cell types [124]. Given this model, the question of whether transcription factory formation is a byproduct of the process of transcription, or whether these are stable structures whose formation, in fact, precedes and/or drives transcription itself, remains unanswered. What is clear, however, is that the colocalization of genomic loci into these “factories” is a strongly tissue-specific mark of both chromatin folding and 3D genome organization in the nucleus.

### Nucleolar associating domains

The nucleolus is the largest subnuclear organelle in the nucleus of eukaryotic cells and is the prototype for transcription factories, as it serves as the primary site of rRNA biogenesis. In addition to its primary role as the site of rRNA transcription and maturation, the nucleolus also hosts several other biological processes, including viral replication, signal recognition particle biosynthesis, and sequestration of proteins (reviewed in [126]). Nucleoli assemble around the rDNA genes clustered from different chromosomes, where the genes are transcribed by RNAPI. In a given nucleus, only a subset of rDNA loci are transcribed at once, where they are looped into the nucleolus. The remaining rDNA loci are located at the periphery of the nucleolus to form constitutive heterochromatin. Genomic regions that interact frequently with the nucleolus are called nucleolar associating domains (NADs) [49], [127]. NADs are characterized by repetitive DNA elements, mostly from centromeric and pericentromeric regions, are gene poor, and typically contain silent chromatin (*e.g.*, regions of the inactive X chromosome (Xi), repressed olfactory receptor genes, tissue-specifically repressed RNAPII genes), and several RNAPIII-transcribed genes. NADs cover about 4% of the human genome and are significantly overlapped with LADs (discussed in further detail below), indicating that a certain amount of redistribution occurs between the nuclear lamina and nucleolar periphery after mitosis [49], [127]. Mechanisms for this redistribution remain poorly understood, though it has been shown that nucleolus tethering may be mediated by *trans* acting factors such as CTCF, chromatin assembly factor (CAF)-1, nucleolar proteins, and potentially lncRNAs [126].

### LADs

LADs refer to the regions of the genome that interact with the nuclear lamina at the interior of the nuclear envelope. LADs were first characterized using the DamID technique, which has revealed that mammalian LADs are large, gene-poor domains spanning 40 kb–30 Mb and covering ∼40% of the genome [50], [128]. LADs are enriched for heterochromatic silencing marks, largely overlap with the previously-identified H3K9me2 locks, and show very sharp borders that are significantly enriched for bidirectional transcription, CpG islands, and CTCF binding sites [50]. These features are reminiscent of the borders found at self-associating domains. As with NADs, the mechanisms underlying tethering of LADs to the nuclear periphery largely remain unclear. However, a recent single-cell study has revealed that LADs showing stable contact (*i.e.*, contact across many single cells) with the nuclear lamina (NL) are extremely gene poor, suggesting a structural role, whereas LADs with variable NL contacts tend to be cell-type specific [52]. Moreover, the consistency of NL contacts is inversely linked to gene activity in single cells and correlates positively with the heterochromatic histone modification H3K9me3 [52], suggesting that the tethering of LADs to the NL plays an important role in physically and functionally compartmentalizing eukaryotic genomes.

### Chromatin loops and gene regulation

Looping is an intrinsic property of chromatin fibers and serves as the basic mechanism of chromatin folding. In as early as 1878, Walther Flemming observed large chromosomal loops in the so-called lampbrush chromosomes of amphibian oocytes [125]. Ptashne and others have since posited that long-range looping interactions may be key effectors of gene expression [129], a hypothesis that has gained credence, thanks to recent mapping efforts via 3C-based methods. The chromatin loop is likely tightly related to the formation of self-associating domains. For example, a recent work has shown that the stability of a TAD is determined by specific long-range loops within it [130]. The best-studied chromatin loops are those between genes and their distal regulatory elements, such as enhancers. One such example is the observation of an active chromatin hub (ACH) at the active beta- and alpha-globin loci. The ACH configuration is formed when multiple regulatory elements are juxtaposed against one another in 3D space via looping to coordinate gene expression [131].

Recent genome-wide mapping of chromatin interactions has uncovered general features of this type of loop. First, ∼50% of active genes are engaged in long-range chromatin interactions in the cell types examined [132], [133]. Notably, those active genes that are not found to interact with a distal enhancer are enriched in housekeeping genes [132]. Second, in addition to promoter–enhancer interactions, promoter–promoter and enhancer–enhancer loops have also been detected, and there is extensive co-localization among multiple promoters and/or multiple distal-acting enhancers [133], [61], [134], [135]. Given that 3C-based methods are designed to detect second order interactions (*i.e.*, pairs of interacting loci), the question remains whether an element interacts with multiple other elements simultaneously within the same nuclear environment, or whether these interactions actually occur within different single cells. As discussed in further detail below, arriving at an answer to these questions may become possible through the further development of single-cell epigenomic technologies. Third, promoter–enhancer interactions generally show high cell type specificity and are correlated with cell type-specific transcription [133], [61], [134], [135], though it has been argued that promoter–enhancer loops are generally unchanged across tissue contexts and across development [132], [136]. Collectively, these findings nonetheless underscore that chromatin looping is an important mechanism by which long-range interaction between distal regulatory elements and genes may be achieved.

Building upon these findings, recent functional studies using gene editing tools have further suggested a causal link between chromatin looping and gene regulation. It has long remained unclear whether looped interactions are a prerequisite for or merely a consequence of gene regulation. Direct evidence has been obtained recently, demonstrating that chromatin looping between a gene promoter and a strong enhancer can lead to transcriptional activation [137], [138]. The Blobel group, in collaboration with synthetic ZFN pioneers Sangamo Biosciences, recently showed that chromatin loops may be induced between the globin locus control region (LCR) and the beta-globin promoter in GATA1 knock-out murine cells using synthetic zinc-finger proteins tethered to the self-association domain of Ldb1. These induced chromatin loops led to substantial activation of ß-globin transcription in the absence of GATA1 [137]. Using the same approach, the group also more recently demonstrated that forced LCR–promoter looping could lead to transcriptional reactivation of the developmentally-silenced fetal ϒ-globin gene in adult murine erythroblasts [138]. These new insights argue that, in the proper context, forced chromatin looping can directly guide transcriptional activity [99], [139].

Many factors, including transcription factors (*e.g.*, CTCF, YY1, and NRSF), co-activators (*e.g.*, mediators), chromatin structural proteins (*e.g.*, cohesin), and ncRNAs (*e.g.*, Xist [140], Firre [141], [142], and HOTTIP [143]) have been shown to play roles in mediating chromatin looping. The roles of CTCF and cohesin in spatial genome organization are by far the best characterized. Both CTCF and cohesin have been found to bind thousands to tens of thousands genomic sites, a significant portion of which are co-occupied by both proteins in mammalian cells [18], [125], [144]. Early studies also established CTCF as a transcription factor with versatile roles in transcription activation and repression, as well as a global insulator protein [145], [146]. Cohesin is best known for its role in sister chromatid cohesion, chromosome segregation, and DNA repair [147]. Insights obtained from recent studies have also suggested that CTCF and cohesin play important roles in the hierarchical folding of the interphase chromosome, from chromatin looping to establishment of chromatin domains. It has been found that CTCF mediates thousands of chromatin loops in mouse and human genomes, which account for a substantial portion of all the loops detected in a genome [62], [71], [105], [148], [149], [150]. The formation of CTCF-mediated loops requires cohesin, which also co-localizes with mediators to facilitate tissue-specific promoter–enhancer looping [151]. Moreover, it has been revealed that the orientation of CTCF binding guides directional chromatin looping [62], [96], [98], [152]. This is in agreement with an extrusion model of loop formation [98], [153].

It is believed that CTCF and cohesin also play important roles at the chromatin-domain level. CTCF binding has been found enriched at LAD boundaries, suggesting involvement in the formation of LADs [50]. CTCF and cohesin are also enriched at the boundaries of TADs, and depletion of cohesion and CTCF results in widespread changes in topological organization [154]. As mentioned briefly above, this was also shown recently by a study demonstrating that *IDH* mutations promote gliomagenesis by disrupting CTCF binding via hypermethylation, in turn disrupting TAD boundaries and allowing aberrant enhancer–promoter interactions to activate normally-insulated oncogenes [121]. These results suggest a general role for CTCF and cohesin in chromatin folding and genome compartmentalization.

## Future directions

The synthesis of classical microscopy-based approaches and more recent high-throughput biochemical techniques has led to an explosion in our knowledge of the physical organization of eukaryotic genomes. Through a diverse array of techniques including electron microscopy, FISH, ChIP-seq, DamID, as well as 3C and its derivatives, we are generating increasingly fine-scale catalogs of the chromatin loops, self-associating domains, and CTs that comprise the eukaryotic nuclear genome. Given this dense catalog of structural elements, then, we believe that the field will eventually move into two primary directions: (i) functional dissection of this vast catalog of structural elements, and (ii) large-scale characterization of the dynamics and mechanisms of chromatin folding both across biological processes such as differentiation, and across homogenous and heterogeneous cell populations.

### Functional dissection of structural elements

The advent of CRISPR/Cas9 as an easy to use, highly-multiplexable system for perturbing primary sequence has opened up considerable avenues to testing the functional significance of genomic elements. We predict the continued use of genome editing reagents in validating key structural elements (*e.g.*, CTCF binding sites), with respect to various phenotypes of interest (*e.g.*, pathogenicity and dysregulation of global and local gene expression). Already, several groups have successfully utilized Cas9-mediated genome editing to generate clonal populations harboring inverted or deleted transcription factor binding sites, and have performed assays like Hi-C and RNA-seq to link structural and functional changes [94], [96], [98].

As low-throughput (*e.g.*, test of single edited clones) approaches become more popular, we anticipate the eventual development of high-throughput screens for large-scale characterization of structural elements. Already, genome editing-based lentiviral and *in vivo* saturation mutagenesis screens have been employed, to dissect the functional significance of genes [155], [156], [157], codons [158], small insertions/deletions (indels) [159], [160], and SNPs [158]. A key next step in determining the functional significance of cataloged elements will be employing such approaches to perturb key structural features in a variety of biological contexts; these experiments may be critical to eventually understanding the link between human disease phenotypes (*e.g.*, cancer) and dysregulation of chromatin architecture.

### Characterizing structural dynamics across time and space

Questions regarding the dynamics of chromatin—the processes by which chromatin architecture and state change as a function of a given biological process—remain largely unanswered. The nascent field of single-cell epigenomics [161], however, has offered a key set of tools that may finally be able to address such questions. While traditional epigenomic assays must be performed on populations of cells, single-cell epigenomics provide an opportunity to characterize heterogeneity within populations—an invaluable tool for both defining novel cell types from a heterogenous population (*e.g.*, an organ system), and for characterizing transitory states in biological processes such as differentiation. Recently-published approaches such as single-cell DamID [52], single-cell ChIP-seq [88], and single-cell Hi-C [60] all provide valuable proof-of-concept for such assays. The next step, then, is to scale these approaches to easily process hundreds of thousands of single cells. We recently described a method that leverages combinatorial DNA barcoding of single cells to provide chromatin accessibility information from thousands of cells in a single experiment [162]. Such approaches may be adapted to other epigenomic assays, including Hi-C, DamID, and ChIP, thus providing a way forward to achieving the required throughput to confidently define new cell types, or organize populations of cells going through some biological process into some sort of “pseudotime.”

It may also be useful to consider the marriage of single-cell biochemical techniques with complimentary microscope-acquired *in situ* transcriptomic datasets [163], [164], [165], [166]. *In situ* transcriptomics may, for example, be necessary to properly spatially organize large populations of tissue-derived nuclei in some biologically meaningful way. Furthermore, by matching *in situ* transcriptomic data with replicate single-cell epigenomic experiments in this way, one may be able to link differential genome architectural features with gene regulatory phenomena, thus furthering our progress toward ultimately understanding the links between 3D genome architecture and gene regulation.

Of course, the application and development of any of these techniques is intertwined with the development of data analytical techniques. While algorithmic development will have to keep pace with the development of these technologies, we believe that incredible strides already made in the relatively young field of single-cell RNA sequencing [167] are a positive indicator that analytical methods will be able to keep pace with this exploding field.

## Closing remarks

There are many fundamental and long-standing biological questions linked to 3D genome architecture, and we close by echoing a handful of them below. Does genome architecture itself define cellular identity? How does chromatin state (*i.e.*, histone modifications and DNA methylation) impact higher-order chromatin structure? How might defects or differences in 3D genome architecture lead to human disease? Obtaining the knowledge necessary to answer these questions requires a multi-pronged approach employing creative microscopic, biochemical, and computational tools. As reviewed here, these are thankfully requirements that the field is actively addressing, suggesting that we will be well-positioned to answer many if not all of these pressing questions in the years to come.

## Competing interests

The authors have declared that no competing interests exist.

## Figures and Tables

**Table 1:** Biochemical tools for probing genomic interactions

**Interaction type**	**Methods**	**Nuclear landmarks**	**Refs.**
DNA–protein	ChIP	NPC	[47]
	DamID	NPC and nuclear lamina	[48], [50], [51], [52]
	Sedimentation Fractionation	Nucleolus	[49], [127]

DNA–RNA	ChIRP	–	[53]
	CHART	–	[54]
	RAP	–	[55]

DNA–DNA	3C and derivatives	–	[57]

*Note:* ChIP, chromatin immunoprecipitation; NPC, nuclear pore complex; DamID, DNA adenine methyltransferase identification; ChIRP, chromatin isolation by RNA purification; CHART, capture hybridization analysis of RNA targets; RAP, RNA antisense purification; 3C, chromosome conformation capture.

**Table 2:** The 3C family

**Scale**	**Method**	**Main features**	**Refs.**
Whole-genome	Hi-C	For mapping whole-genome chromatin interactions in a cell population; proximity ligation is carried out in a large volume	[58]
	TCC	Similar to Hi-C, except that proximity ligation is carried out on a solid phase-immobilized proteins	[59]
	Single-cell Hi-C	For mapping chromatin interactions at the single-cell level	[60]
	DNase Hi-C	Chromatin is fragmented with DNase I; proximity ligation is carried out in solid gel	[61]
	*In situ* Hi-C	Proximity ligation is carried out in the intact nucleus	[62]
	Micro-C	Chromatin is fragmented with micrococcal nuclease	[64]
	*In situ* DNase Hi-C	Chromatin is fragmented with DNase I; proximity ligation is carried out in the intact nucleus	[63]

Targeted	3C	The founding method of the 3C family of techniques; for detecting chromatin interactions between a pair of genomic loci	[57]
	ChIP-loop	Combines 3C with ChIP; for detecting chromatin interactions mediated by a particular protein between a pair of genomic loci	[65]
	4C	For detecting chromatin interactions between one locus and the rest of the genome	[66], [67]
	e4C	A more sensitive version of 4C by replacing inverse PCR with primer extension	[68]
	5C	For detecting chromatin interactions between multiple selected loci	[69]
	ChIA-PET	For detecting genome-wide chromatin interactions mediated by a particular protein	[70], [71]
	Capture-C	Combines 3C with a DNA capture technology; equivalent to a high-throughput version of 4C	[72]
	Capture-Hi-C	Combines Hi-C with a DNA capture technology; equivalent to a high-throughput version of 4C	[73]
	Targeted DNase Hi-C	Combines DNase or *in situ* DNase Hi-C with a DNA capture technology; equivalent to a high-throughput version of 4C	[61]

*Note:* 3C, chromosome conformation capture; TCC, tethered conformation capture; 4C, circularized chromosome conformation capture; e4C, enhanced 4C; 5C, carbon-copy chromosome conformation capture; ChIA-PET, chromatin interaction analysis by paired-end tag sequencing.
